# High-Pressure Synthesis of Pd(N_5_)_2_·N_2_: One-Dimensional Pentazolate Coordination Polymer with Guest Nitrogen Molecules

**DOI:** 10.1021/acs.inorgchem.6c03152

**Published:** 2026-09-28

**Authors:** Andrey Aslandukov, Alena Aslandukova, Elena Bykova, Stella Chariton, Vitali Prakapenka, Jesse S. Smith, Mohammad Mahmood, Alexander F. Goncharov, Maxim Bykov

**Affiliations:** † 425797 Goethe University Frankfurt, Institute of Inorganic and Analytical Chemistry, Max-von-Laue-Straße 7, Frankfurt 60438, Germany; ‡ 9173 Goethe University Frankfurt, Institute of Geosciences, Altenhöferallee 1, Frankfurt 60438, Germany; § Center for Advanced Radiation Sources, 189299 University of Chicago, Lemont, Illinois 60439, United States; ∥ HPCAT, X-ray Science Division, Argonne National Laboratory, Lemont, Illinois 60439, United States; ⊥ Department of Mathematics, Howard University, Washington D.C. 20059, United States; # The Earth and Planets Laboratory, 1485 Carnegie Institution for Science, Washington D.C. 20015, United States

## Abstract

Pentazolates, an emerging family of nitrogen-rich materials featuring the aromatic cyclo-N_5_^–^ anion, typically crystallize as nonpolymeric or three-dimensional (3D) coordination networks, whereas low-dimensional (1D and 2D) forms remain exceptionally rare. Here, we exploit the square-planar coordination preference of *d* ^8^ Pd^2+^ to achieve dimensional control and synthesize Pd­(N_5_)_2_·N_2_, the first high-pressure 1D pentazolate coordination polymer. The compound forms in a laser-heated diamond anvil cell at 118 GPa and 2900 K, while lower-pressure experiments yield only PdN_2_ dinitride. The crystal structure of Pd­(N_5_)_2_·N_2_ was determined by synchrotron single-crystal X-ray diffraction from a multigrain sample and corroborated by density functional theory calculations. This discovery establishes a strategy for engineering low-dimensional pentazolate-based materials and potentially enabling dimensional control in other polynitrides. We also provide an overview of experimentally synthesized high-pressure pentazolates and discuss how cation properties influence their synthesis pressures, thereby improving understanding of pentazolate formation pressures.

## Graphical abstract

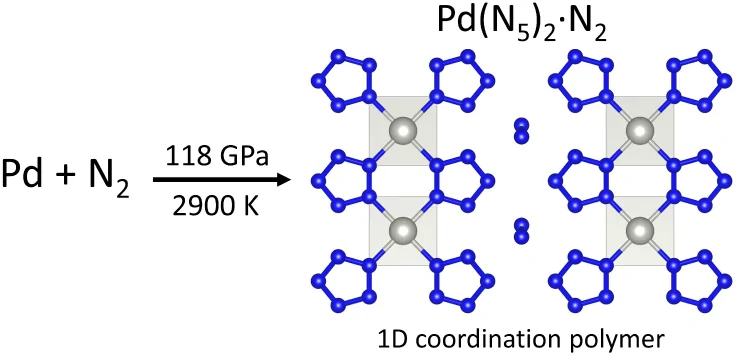

## Introduction

Synthesis of nitrogen-rich materials represents a modern challenge due to their potential use as environmentally friendly high-energy-density materials. One of the advances in this field is the discovery of the pentazolate anion (N_5_^–^), which consists solely of nitrogen atoms and whose aromaticity imparts relative stability to pentazolate-based compounds. Since the first stabilization of the N_5_^–^ anion in 2017,^1,2^ numerous metal pentazolate salts featuring isolated N_5_^–^ units have been synthesized,^3–7^ and many of them contain substantial amounts of coordinated water that stabilize the anion via hydrogen bonding. Solvent-free pentazolates have been obtained either via a conventional chemical route by salt-exchange reactions—yielding compounds such as AgN_5_,^7^ Cu­(N_3_)­(N_5_),^7^ LiN_5_,^8^ KN_5_,^9^ and NH_4_N_5_ ^9^ or by high-pressure synthesis, which until recently has produced only pentazolates of monovalent cations (e.g., LiN_5_, NaN_5_, NaN_5_·N_2_, K_9_N_56_, CsN_5_, AgN_5_).^2,10–16^ In 2025, the first high-pressure trivalent-cation pentazolate, Y­(N_5_)_3_·N_2_, containing three pentazolate anions per metal center, was synthesized at 125 GPa.^17^

Notably, most pentazolates reported to date either are nonpolymeric or crystallize as three-dimensional (3D) coordination polymers.^2,7–18^ Low-dimensional (1D and 2D) coordination polymers are important because their reduced dimensionality gives rise to distinct structural, electronic, magnetic, optical, and mechanical properties that are not achievable in 3D extended structures. However, control over the dimensionality of the pentazolate compounds remains elusive.

Group 10 cations Ni^2+^, Pd^2+^, and Pt^2+^ possess *d* ^8^ electron configuration, which stabilizes the square coordination of these ions, which can be used for the targeted design of low-dimensional (1D and 2D) coordination polymers. Binary Ni–N, Pd–N, and Pt–N systems have been studied up to moderate pressures (40–60 GPa), and NiN_2_,^19,20^ PdN_2_,^21^ and PtN_2_ ^22^ dinitrides have been discovered. PdN_2_ and PtN_2_ crystallize in a pyrite-type structure,^21,22^ while NiN_2_ adopts a marcasite-type structure^19^ and upon decompression undergoes a phase transition to layered *P*4*/mbm*-NiN_2_, demonstrating a pentagonal 2D Cairo-type tessellation.^20^ Polynitrides of Ni, Pd, and Pt are yet unknown, although Ni­(N_5_)_2_ and Pd­(N_5_)_2_—2D pentazolate coordination polymers—are predicted.^23^

Here, we report the high-pressure high-temperature synthesis and characterization of Pd­(N_5_)_2_·N_2_ at 118(2) GPa. The crystal structure of Pd­(N_5_)_2_·N_2_ has a unique topology and represents the first high-pressure 1D pentazolate coordination polymer with guest nitrogen molecules.

## Results and Discussion

In the present study, the diamond anvil cell loaded with a palladium foil piece embedded in nitrogen was used (Figure S1). Laser heating of the sample was performed at several pressures (Table 1). Moderate laser heating of the sample at 102(2) and 105(2) GPa yields a single product: pyrite-type PdN_2_ (Figure 1a), previously known at lower pressures.^21^ Its crystal structure at 102(2) GPa was unambiguously determined by single-crystal X-ray diffraction from a multigrain sample. Further structural details on PdN_2_, including its behavior at 118(2) GPa and upon decompression, as well as insights from density functional theory, are provided in the Supporting Information.

**Table 1:** Summary of High-Pressure High-Temperature Experiments

Pressure, GPa	Temperature, K	Reaction products
102(2)	1950(300)	PdN_2_
105(2)	1800(300)	PdN_2_
118(2)	2900(300)	PdN_2_, Pd(N_5_)_2_·N_2_

**Figure 1:**
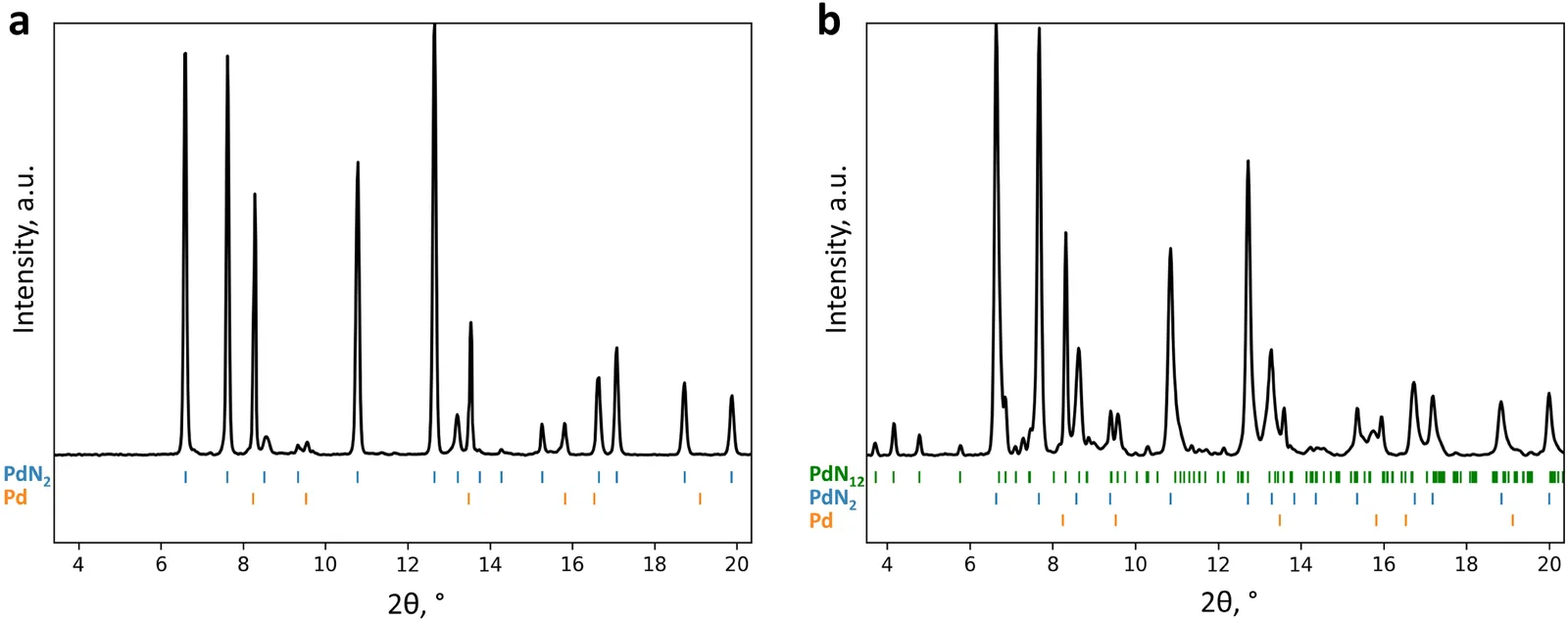
Powder X-ray diffraction patterns (λ = 0.2952 Å) of the laser-heated sample at (a) 105(2) GPa and (b) 118(2) GPa. Orange ticks indicate the reflection positions of Pd, blue ticks correspond to PdN_2_, and green ticks to Pd­(N_5_)_2_·N_2_ (denoted as PdN_12_ in the figure). Structure refinements and phase identification were based on single-crystal data sets.

Further laser-heating of a mixture of Pd, PdN_2_, and nitrogen at 118(2) GPa and a noticeably higher temperature resulted in the formation of another crystalline phase (Figure 1b). Synchrotron single-crystal X-ray diffraction from a multigrain sample revealed its stoichiometry and crystal structure (Figure 2, Figure S2). The composition is Pd­(N_5_)_2_·N_2_ with a remarkably high nitrogen-to-metal ratio 12:1. The novel phase crystallizes in an orthorhombic space group *Pnnm* (#58) with the lattice parameters *a* = 3.840(2) Å, *b* = 4.557(12) Å, *c* = 9.089(7) Å at 118(2) GPa (Table S1). The refinement against single-crystal X-ray diffraction data resulted in very good agreement factors for the data collected at higher than a megabar pressure (Table S1). The formation of Pd­(N_5_)_2_·N_2_ may be promoted by both increasing pressure and temperature, however, the individual contributions of these parameters cannot be determined from the available data.

**Figure 2:**
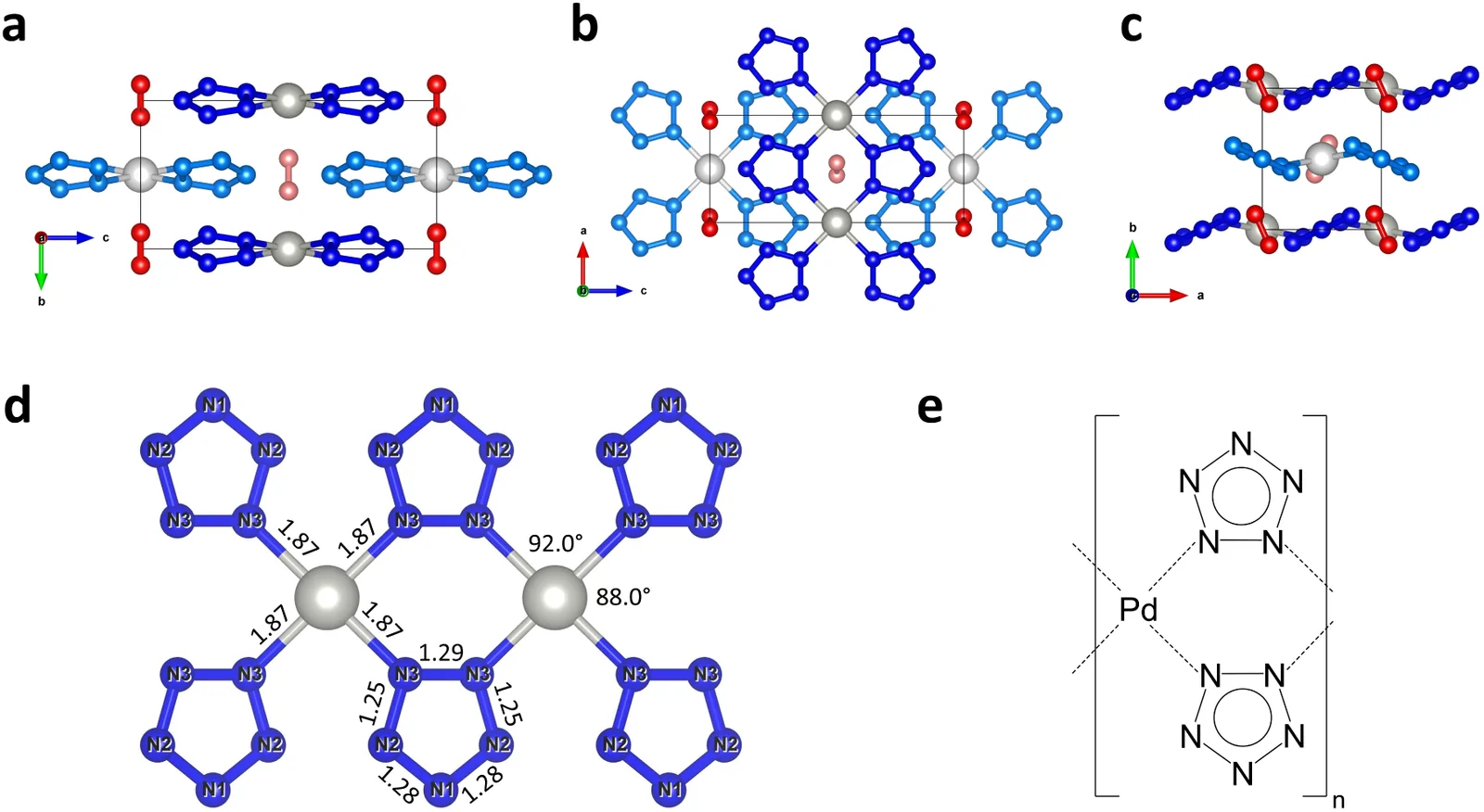
Crystal structure of Pd­(N_5_)_2_·N_2_ viewed along (a) *a* direction, (b) *b* direction, and (c) *c* direction. Gray balls correspond to palladium atoms, blue balls correspond to nitrogen atoms of pentazolate rings, and red balls—to nitrogen atoms of nitrogen dimers. The darker and lighter color scheme highlights the atoms in the two layers, symmetry-related by the *n* glide plane. (d) Geometry of Pd­(N_5_)_2_ ribbons viewed along the *b*-direction with indicated Pd–N distances, ∠N–Pd–N angles, and N–N bond lengths within N_5_^–^ rings. (e) Schematic representation of the one-dimensional coordination palladium–pentazolate polymer.

The crystal structure of Pd­(N_5_)_2_·N_2_ is shown in Figure 2. Pd occupies one atomic position, while N occupies four distinct atomic positions. There are two distinct types of nitrogen species in the crystal structure: pentazolate rings and dimers. Pentazolate rings are tilted with respect to the *ac* plane (Figure 2a,c). Palladium atoms in Pd­(N_5_)_2_·N_2_ are coordinated by four pentazolate rings, possessing square coordination typical for group 10 metals. The structure in general can be described as a two-layer stacking along the *b* direction. These two layers are symmetry-related by the *n* glide plane. Each layer contains Pd­(N_5_)_2_ ribbons (Figure 2d,e)—1D coordination polymers—running along *a*-direction separated by N_2_ dimers. Pd­(N_5_)_2_·N_2_ has a novel structure type with a topology of distorted LiPd_2_Tl,^24^ if pentazolate rings and nitrogen dimers are replaced by their centers according to the recently proposed approach for structural typification of inorganic solvent-free pentazolates.^17^

The average bond length in pentazolate rings in Pd­(N_5_)_2_·N_2_ is 1.268(6) Å at 118(2) GPa (Figure 2d), which agrees well with the bond lengths in pentazolate anion at a similar pressure.^17^ The nitrogen–nitrogen bond length in the dimer is 1.09(5) Å, consistent with a triple N≡N bond, indicating that this is a nitrogen molecule inclusion. The chemical formula Pd­(N_5_)_2_·N_2_ suggests that palladium is in its typical oxidation state +2, making Pd­(N_5_)_2_·N_2_ the first high-pressure pentazolate of a divalent cation.

Notably, at 118 GPa, Pd^2+^ still prefers a coordination number CN = 4 with square coordination—and not to increase its CN that might be expected according to high-pressure coordination-number rule^25^—highlighting the extreme stability of square coordination for *d* ^8^ cations along a wide pressure range.

Pd­(N_5_)_2_·N_2_ is a pentazolate-incorporating guest nitrogen molecules, expanding the family of nitrogen-inclusion pentazolate compounds alongside NaN_5_·N_2_ ^14^ and Y­(N_5_)_3_·N_2_.^17^ Notably, previously synthesized high-pressure pentazolates typically exhibit 3D metal–pentazolate coordination networks. In contrast, Pd­(N_5_)_2_·N_2_ is the first example of a high-pressure 1D pentazolate coordination polymer. Thus, Pd­(N_5_)_2_·N_2_ represents a classical 1D coordination network based on metal–nitrogen linkages.^26^ Five common topological types are known for such 1D coordination-polymer chains.^26^ Pd­(N_5_)_2_·N_2_ features a ribbon-type chain, enabled by the μ_2_-η^1^(N1):η^1^(N2) coordination mode of the pentazolate ring (Figure 3a).

**Figure 3:**
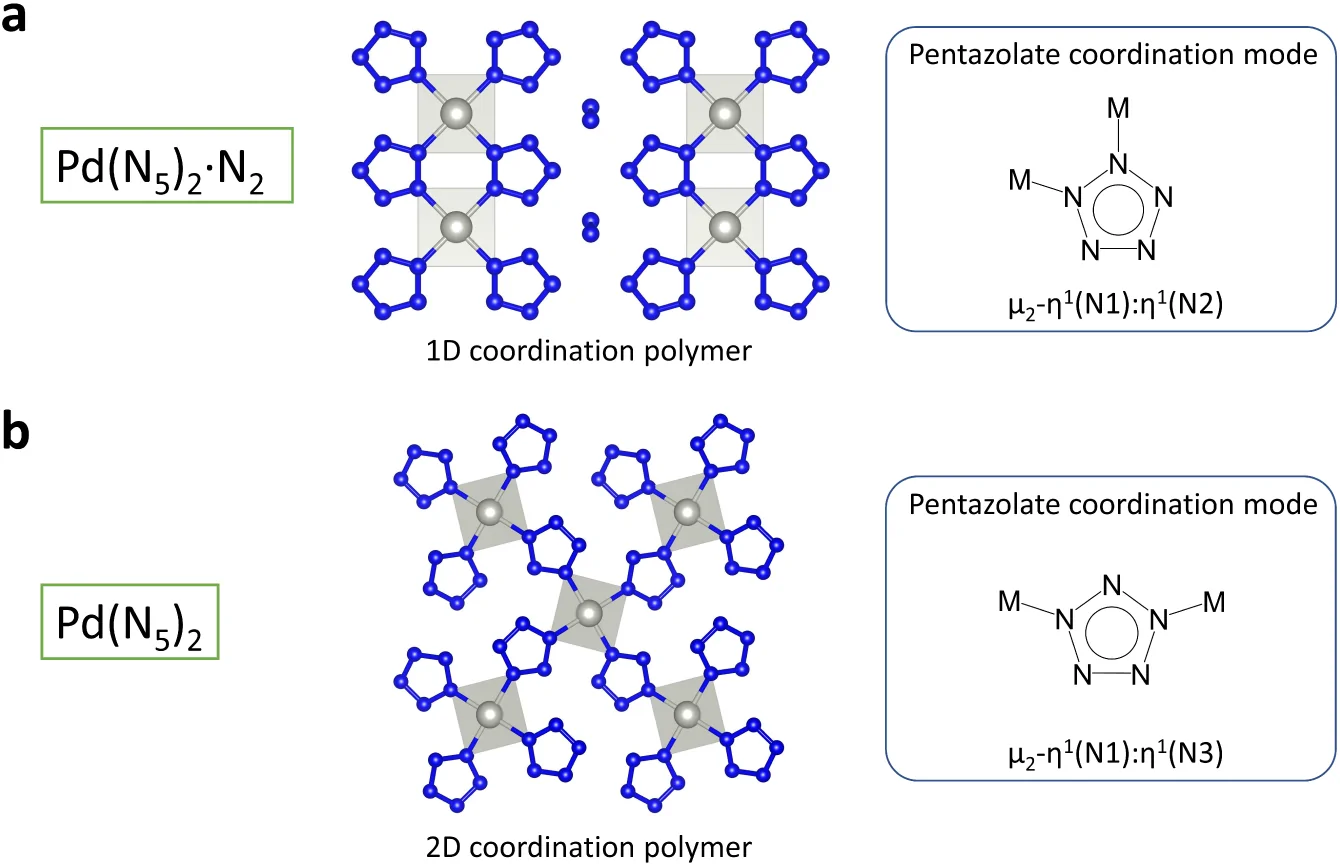
Comparison of (a) here-synthesized Pd­(N_5_)_2_·N_2_ with (b) predicted Pd­(N_5_)_2_.^23^

Among numerous theoretical predictions of metal pentazolates, there is only one mention of palladium pentazolate in the literature: a monoclinic Pd­(N_5_)_2_ stabilized at 50 GPa was found by Huang et al. using a structural search.^23^ In the predicted Pd­(N_5_)_2_, palladium also possesses square coordination, and each pentazolate ring is coordinated by two palladium atoms; however, the coordination mode of the pentazolate anion is μ_2_-η^1^(N1):η^1^(N3) compared to μ_2_-η^1^(N1):η^1^(N2) in Pd­(N_5_)_2_·N_2_. This results in a drastic difference in the structural motif: predicted Pd­(N_5_)_2_ represents a 2D coordination polymer, while here-synthesized Pd­(N_5_)_2_·N_2_ is a 1D coordination polymer (Figure 3).

On decompression at ambient temperature, Pd­(N_5_)_2_·N_2_ was observed down to 64(2) GPa (Table S2). As expected from the crystal structure, the compressional behavior is anisotropic: the *a* axis is the least compressible, as it coincides with the direction of the Pd­(N_5_)_2_ ribbons, whereas the *b* axis—perpendicular to the ribbons—is the most compressible (Figure 4a).

**Figure 4:**
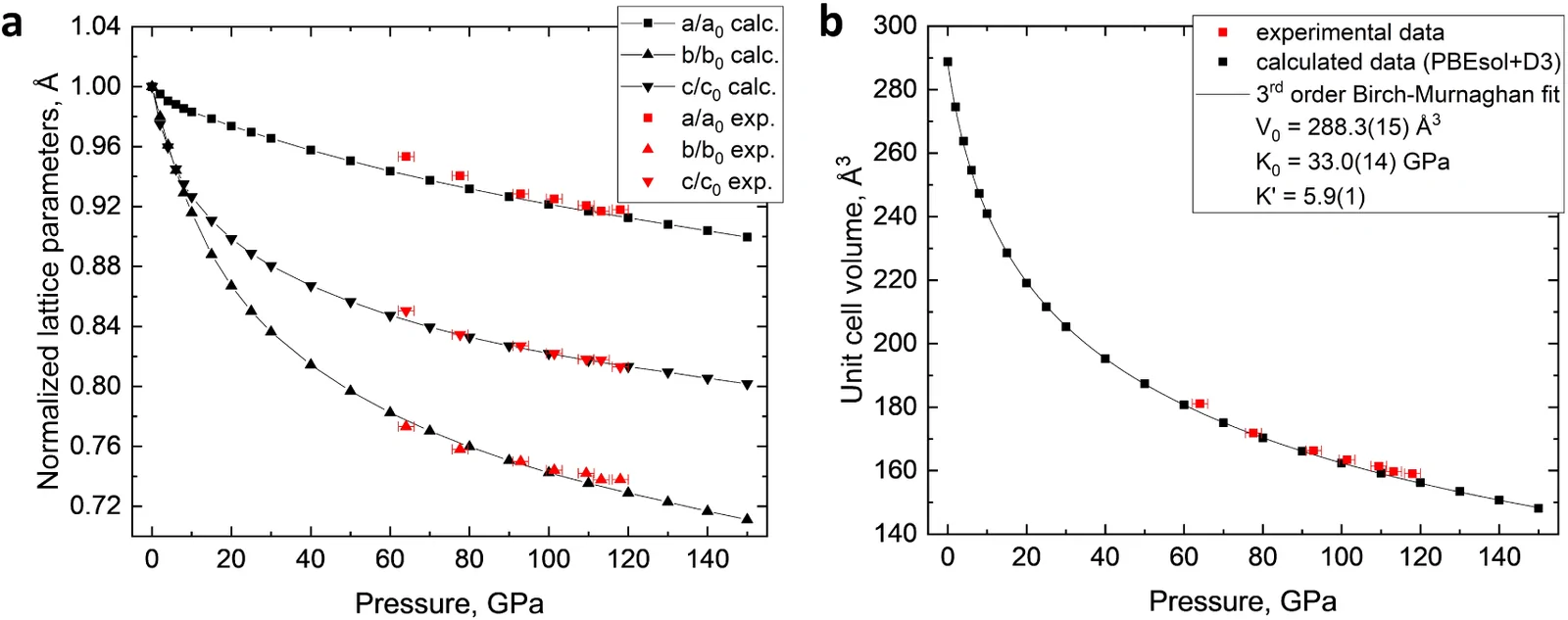
Pressure dependence of (a) normalized lattice parameters and (b) unit cell volume for Pd­(N_5_)_2_·N_2_.

For cross-validation of the Pd­(N_5_)_2_·N_2_ crystal structure and to get a deeper insight into the stability and physical properties of the novel compound, we performed density functional theory (DFT) calculations (see the Experimental and Computational Section for details). We carried out variable cell structural relaxation for Pd­(N_5_)_2_·N_2_ at 118 GPa and found that the relaxed unit cell volume and unit cell parameters closely reproduce the corresponding experimental values (Table S3). The calculated bond lengths are also in good agreement with the experimental data (Table S3).

The bonding analysis based on integrated crystal orbital bond index (ICOBI)^27^ values (Table S4) indicates an appreciable covalent contribution to the Pd–N3 bonding, as expected for transition-metal compounds. The charge distribution analysis (Table S5) reveals a small negative charge on the N_2_ dimers, indicating a slight charge transfer to the N_2_ molecules within the crystal structure.

The phonon dispersion relations calculated in the harmonic approximation show that Pd­(N_5_)_2_·N_2_ is dynamically stable at a synthesis pressure of 118 GPa (Figure S3a). The remarkable feature of the phonon dispersions is the separated high-frequency optic modes (at ∼2500 cm^–1^), which correspond to the vibrations of noncharged triple-bonded nitrogen dimers. The same characteristic phonon density-of-states feature is demonstrated by other nitrogen-inclusion polynitrides, i.e., Y­(N_5_)_3_·N_2_, ReN_8_·xN_2_, WN_8_·N_2_, Hf_4_N_20_·N_2_, and Os_5_N_28_·3N_2_.^17,28,29^ DFT calculations indicate that Pd­(N_5_)_2_·N_2_ is a semiconductor (Figure S3b) as expected for pentazolates.^17^

To trace the structural behavior at lower pressures, the full variable-cell structure relaxations for Pd­(N_5_)_2_·N_2_ were performed between 0 and 150 GPa. PBEsol+D3 calculations suggest that the initial Pd­(N_5_)_2_·N_2_ structure can be relaxed without significant structural changes down to ambient pressure, although the structure is dynamically unstable at 1 bar (Figure S4). The P–V data agree well with the experimental decompression data. The volume-pressure data of DFT-relaxed structures of Pd­(N_5_)_2_·N_2_ were fitted with a 3rd-order Birch-Murnaghan equation of state (Figure 4b), leading to the bulk modulus of K_0_(Pd­(N_5_)_2_·N_2_) = 33.0(14) GPa.

All high-pressure pentazolates reported to date are summarized in Figure 5 and Table S6. It should be noted that the available experimental data remain quite limited, and results based on structural searches combined with powder X-ray diffraction are generally less reliable than those obtained from direct structure determination using single-crystal X-ray diffraction from multigrain samples. The pressure required for the synthesis of pentazolates with different cations may depend on several parameters, including the cation’s ionic radius, oxidation state, and electronegativity. Although these parameters are interrelated, we discuss below how each factor may influence the required synthesis pressure based on the available data.

**Figure 5:**
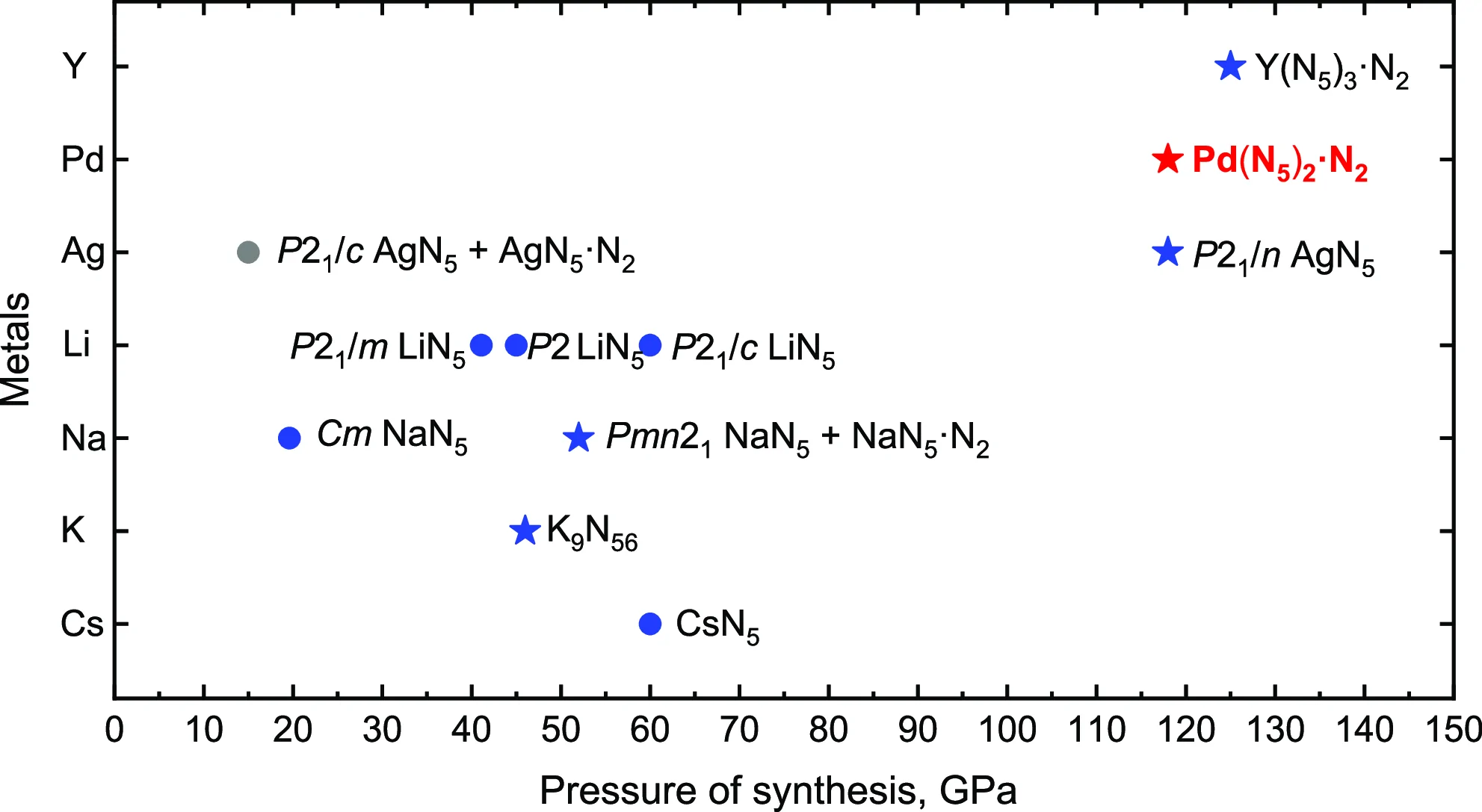
Summary of all experimentally synthesized high-pressure pentazolates reported so far. The cations are sorted first by oxidation state and then by electronegativity. Circle symbols correspond to compounds whose crystal structures were found via structural search and can explain the powder X-ray diffraction patterns; star symbols correspond to compounds whose crystal structures were directly solved via single-crystal X-ray diffraction from a multigrain sample. The gray circle corresponds to AgN_5_ and AgN_5_·N_2_ claimed by Zhang et al.,^30^ however those data were recently refuted by Liang et al.,^16^ and therefore, these compounds are not considered in the data interpretation.

### Ionic Radius

According to the general high-pressure rule^25^ within a given group of the periodic table, smaller cations at elevated pressures tend to behave similarly to larger cations at lower pressures. For example, NaCl and KCl under pressure adopt the crystal structure of CsCl;^31^ Ln_2_O_3_ oxides of heavier lanthanides undergo phase transitions, forming structures that are stable for lighter lanthanides under ambient conditions.^32^ Consequently, for example, one might expect isostructural alkali-metal pentazolates containing smaller cations to form at higher pressures. However, no isostructural pentazolates have been reported to date. The influence of ionic radius is not clearly evident from the limited experimental data (Figure 5), suggesting that other factors may play a more significant role.

### Oxidation State

Notably, most high-pressure pentazolates of monovalent cations form at moderate pressures (20–60 GPa), whereas pentazolates of di- and trivalent cations require pressures above 100 GPa (Figure 5). It is well established that increasing pressure raises the chemical potential of nitrogen, thereby promoting the stabilization of nitrogen-rich MN_*x*_ phases with higher nitrogen content (*x*) under high-pressure conditions.^33^ The formation of pentazolates with di- and trivalent cations requires minimum nitrogen-to-metal ratios of *x* = 10 and *x* = 15, respectively. Therefore, their synthesis can be achieved only at substantially higher pressures compared to monovalent cations, which require a minimum nitrogen content of *x* = 5.

### Electronegativity

The effect of electronegativity is reasonable to compare for cations with the same oxidation state. Notably, AgN_5_ is observed only above 118 GPa,^16^ whereas alkali metal pentazolates form at much lower pressures (20–60 GPa), highlighting the role of electronegativity among monovalent cations: the less electronegative alkali metals stabilize pentazolates far more readily than silver (Figure 5). A plausible explanation is the higher degree of ionicity in alkali metal pentazolates. In these predominantly ionic salts, the negative charge is largely delocalized on the N_5_^–^ anion, preserving its aromatic stabilization, which enhances intrinsic stability and favors formation. In contrast, pentazolates of transition metals contain a covalent component in the metal–pentazolate interaction, which reduces charge transfer to the ring and may make their formation less favorable.

The discussed trends should be interpreted with caution, as they are based on very limited data obtained using methods with different levels of reliability. Nevertheless, based on the factors discussed above, one may expect pentazolates of tetravalent (4+) cations to form only at pressures above 125 GPa. In contrast, alkaline-earth pentazolates are likely to form at intermediate pressures—between those required for alkali metal pentazolates and for Pd­(N_5_)_2_·N_2_—approximately in the 60–100 GPa range.

## Conclusions

To summarize, in this study, a binary Pd–N system was studied at megabar pressures. Besides PdN_2_ dinitride, a novel binary nitrogen-rich compound Pd­(N_5_)_2_·N_2_ was synthesized in a laser-heated diamond anvil cell at 118 GPa and 2900 K. The crystal structure of Pd­(N_5_)_2_·N_2_ was determined via synchrotron single-crystal X-ray diffraction from a multigrain sample and was additionally corroborated by DFT calculations. Pd­(N_5_)_2_·N_2_ represents the first 1D high-pressure pentazolate coordination polymer. This discovery opens up a route for the targeted design of pentazolates and polynitrides in general in low-dimensional coordination-polymer architectures. A state-of-the-art overview of experimentally synthesized high-pressure pentazolates is presented, and the influence of cation properties on the synthesis pressure is discussed, providing insight into the pressures at which novel pentazolates can form depending on the cation.

## Experimental and Computational Section

### Experiment

The BX90-type large X-ray-aperture DAC^34^ equipped with Boehler-Almax-type diamonds^35^ (culet diameter of 100 μm) was used in the experiment. The sample chamber was formed by preindenting of rhenium gasket to a 20 μm thickness and laser-drilling a hole of 50 μm in diameter in the center of the indentation. A piece of Pd foil (99.95%, Sigma-Aldrich) was placed inside a sample chamber of a BX90 diamond anvil cell. Then, the DAC was loaded with nitrogen that served as a pressure-transmitting medium and as a reagent using a high-pressure gas loading system.^36^ The sample was laser-heated (λ = 1064 nm) using the double-sided laser-heating system of GSECARS (13IDD, APS, Argonne, USA) at several selected pressures and temperatures (Table 1 in the main text). The duration of each laser-heating event was 2 s. The pressure in the DAC was determined monitored by X-ray diffraction of palladium using the equation of state of palladium.^37^

The reaction products contained multiple good-quality single-crystalline domains of novel phases, and they were studied by multigrain synchrotron single-crystal X-ray diffraction at the beamlines GSECARS 13IDD and HPCAT 16IDB (APS, Argonne, USA) with the following beamline setups: GSECARS: λ = 0.2952 Å, beam size ∼3 × 3 μm^2^, Pilatus CdTe 1M detector; HPCAT: λ = 0.4066 Å, beam size ∼3 × 3 μm^2^, Pilatus 1M detector. For the single-crystal XRD measurements, samples were rotated around a vertical ω-axis in a range of ±35°. The diffraction images were collected with an angular step Δω = 0.5° and an exposure time of 2–5 s/frame. For analysis of the single-crystal diffraction data (indexing, data integration, frame scaling, and absorption correction), we used the *CrysAlis* ^*Pro*^ software package.^38^ To calibrate an instrumental model in the *CrysAlis* ^*Pro*^ software, i.e., the sample-to-detector distance, detector’s origin, offsets of goniometer angles, and rotation of both the X-ray beam and the detector around the instrument axis, we used a single crystal of orthoenstatite ((Mg_1.93_Fe_0.06_)­(Si_1.93_, Al_0.06_)­O_6_, *Pbca* space group, *a* = 8.8117(2), *b* = 5.18320(10), and *c* = 18.2391(3) Å). The DAFi program was used for the search for reflection groups belonging to the individual single-crystal domains.^39^ The crystal structures were solved with the SHELXT structure solution program^40^ using intrinsic phasing and refined using ShelXL^41^ in the Olex2 program.^42^ Powder diffraction measurements were performed either without sample rotation (still images) or upon continuous rotation in the range of ±20°ω. The images were integrated to powder patterns using the DIOPTAS software.^43^ Le Bail fits of the diffraction patterns were performed with the Jana2006 software.^44^ Crystal structure visualization was made with the VESTA software.^45^

### Theoretical Calculations

Density functional theory (DFT) calculations were performed using the Projector-Augmented-Wave (PAW) method^46^ as implemented in the Vienna Ab initio Simulation Package (VASP).^47^ The exchange–correlation energy was described within the generalized gradient approximation (GGA) using the Perdew–Burke–Ernzerhof functional revised for solids (PBEsol).^48^ To account for long-range dispersion interactions, the DFT-D3 method with Becke–Johnson damping (D3­(B-J))^49^ was additionally employed for the accurate determination of equilibrium lattice parameters. The PAW potentials with the following valence configurations of 4*d*5*s* for Pd (“Pd”) and 2*s*2*p* for N (“N”) were used. All calculations were performed at 0 K, neglecting both zero-point motion and finite-temperature effects. In geometry optimization, we used a Gamma-centered 14 × 12 × 6 k-mesh, and the plane-wave kinetic energy cutoff was set to 750 eV, with which total energies are converged to better than 2 meV/atom. The electronic convergence criterion was set to Δ*E* = 10^–6^ eV. For Brillouin-zone integrations, the tetrahedron smearing method with Blöchl corrections (ISMEAR = −5) was used.^50^ The phonon frequencies and phonon band-structure calculations were performed in the harmonic approximation with nonanalytical term correction with the help of PHONOPY software^51^ using the finite displacement method for 2 × 2 × 2 supercell. Equation-of-state and static-enthalpy calculations were performed via variable-cell structural relaxations between 1 bar and 150 GPa. The theoretical equation of state of Pd­(N_5_)_2_·N_2_ was obtained by fitting the calculated pressure–volume data using EoSFit7.^52^ Bader charge analysis was done using the algorithm for Bader decomposition of the charge density suggested by G. Henkelman et al.^53^ Mulliken and Löwdin charges as well as crystal orbital bond indices were calculated using the LOBSTER package.^27,54,55^

## Supplementary Material



## Notes

Discussion on PdN_2_, crystallographic information, picture of the DAC sample chamber, phonon dispersion curves and band structure of Pd­(N_5_)_2_·N_2_, charge and chemical bonding analysis for Pd­(N_5_)_2_·N_2_, summary of the reported high-pressure pentazolates, additional references 56,57 (PDF)
