# Supplementary material for: High-Pressure Synthesis of Pd(N5)2·N2: One-Dimensional Pentazolate Coordination Polymer with Guest Nitrogen Molecules
Source: Inorg Chem. 2026 Sep 28;65(40):23573–80. doi: 10.1021/acs.inorgchem.6c03152 (PMC13664098; doi:10.1021/acs.inorgchem.6c03152)
Supplement: Supplementary file 1 [file ic6c03152_si_001.pdf]

Supporting Information

High-pressure synthesis of $Pd(N_5)_2 \cdot N_2$: one-dimensional pentazolate coordination polymer with guest nitrogen molecules

Andrey Aslandukov,[a*] Alena Aslandukova,[a] Elena Bykova,[b] Stella Chariton,[c] Vitali Prakapenka,[c] Jesse S. Smith,[d] Mohammad Mahmood,[e] Alexander F. Goncharov,[f] Maxim Bykov [a*]

[a] Goethe University Frankfurt, Institute of Inorganic and Analytical Chemistry, Max-von-Laue-Straße 7, Frankfurt 60438, Germany

[b] Goethe University Frankfurt, Institute of Geosciences, Altenhöferallee 1, Frankfurt 60438, Germany

[c] Center for Advanced Radiation Sources, University of Chicago, Lemont, Illinois 60439, United States

[d] HPCAT, X-ray Science Division, Argonne National Laboratory, Lemont, Illinois 60439, United States

[e] Department of Mathematics, Howard University, Washington D.C. 20059, United States

[f] The Earth and Planets Laboratory, Carnegie Institution for Science, Washington D.C. 20015, United States

*Correspondence to aslandukov@chemie.uni-frankfurt.de, maxim.bykov@chemie.uni-frankfurt.de

Table of Contents

Supplementary Discussion: PdN_2 3
Supplementary Tables 6
Supplementary Figures 9
References 11

Supplementary Discussion: PdN_2

The mild laser-heating (1800-1950K) of the Pd and N_2 at 102(2) and 105(2) GPa leads to a single product: pyrite-type PdN_2, known at lower pressures[1]. Contrary to the previous study,[1] the crystal structure of PdN_2 at 102(2) GPa was for the first time directly determined *via* single-crystal X-ray diffraction from a multigrain sample (**Table SD1, Fig. SD1**).

After further laser-heating at 118(2) GPa and notably higher temperature of 2900(300) K, PdN_2 was still detected, although its diffraction peaks became substantially broadened. The single-crystal X-ray diffraction pattern can still be indexed with a cubic unit cell, but the lattice angles deviate from 90° considerably, reaching the 3σ level or exceeding it (**Table SD2**), suggesting the formation of a triclinically distorted pyrite-type structure. Such a type of distortion is known in classical pyrite FeS_2.[2]

Table SD1. Structure refinement details of PdN_2 at 102(2) GPa. The full crystallographic data were deposited to the ICSD under the deposition number CSD 2546221.

<table>
<tr><td colspan="2">Chemical formula</td><td colspan="4">PdN_2</td></tr>
<tr><td colspan="2">Temperature (K)</td><td colspan="4">293(2)</td></tr>
<tr><td colspan="2">Pressure (GPa)</td><td colspan="4">102(2)</td></tr>
<tr><td colspan="6">Crystal data</td></tr>
<tr><td colspan="2">Mr</td><td colspan="4">134.42</td></tr>
<tr><td colspan="2">ρ (g/cm^3)</td><td colspan="4">10.173</td></tr>
<tr><td colspan="2">Crystal system, space group</td><td colspan="4">cubic, Pa-3</td></tr>
<tr><td colspan="2">a (Å)</td><td colspan="4">4.444(2)</td></tr>
<tr><td colspan="2">V ($Å^3$)</td><td colspan="4">87.77(12)</td></tr>
<tr><td colspan="2">Z</td><td colspan="4">4</td></tr>
<tr><td colspan="2">Radiation type</td><td colspan="4">X-ray, λ = 0.29521 Å</td></tr>
<tr><td colspan="2">μ (mm^{-1})</td><td colspan="4">11.430</td></tr>
<tr><td colspan="6">Data collection</td></tr>
<tr><td colspan="2">No. of measured, independent and observed [I > 2σ(I)] reflections</td><td colspan="4">167/59/50</td></tr>
<tr><td colspan="2">R_{int}</td><td colspan="4">0.45%</td></tr>
<tr><td colspan="2">$(\sin \theta/\lambda)_{max}$ ($Å^{-1}$)</td><td colspan="4">0.886</td></tr>
<tr><td colspan="6">Refinement</td></tr>
<tr><td colspan="2">$R[F^2 > 4\sigma(F^2)]$, $wR(F^2)$, GOF</td><td colspan="4">2.88%, 10.59%, 1.220</td></tr>
<tr><td colspan="2">data/parameters ratio</td><td colspan="4">59/6</td></tr>
<tr><td colspan="2">$\Delta\rho_{max}$, $\Delta\rho_{min}$ (e $Å^{-3}$)</td><td colspan="4">1.429, -1.455</td></tr>
<tr><td colspan="6">Atomic positions and equivalent isotropic ADPs</td></tr>
<tr><td rowspan="2">Atom</td><td rowspan="2">Wyckoff site</td><td colspan="3">Fractional atomic coordinates</td><td rowspan="2">U_{iso} or U_{eq} ($Å^2$)</td></tr>
<tr><td>x</td><td>y</td><td>z</td></tr>
<tr><td>Pd1</td><td>4b</td><td>0.5</td><td>0.5</td><td>0.5</td><td>U_{eq} = 0.0053(5)</td></tr>
<tr><td>N1</td><td>8c</td><td>0.4124(4)</td><td>0.9124(4)</td><td>0.5876(4)</td><td>U_{eq} = 0.0068(7)</td></tr>
</table>

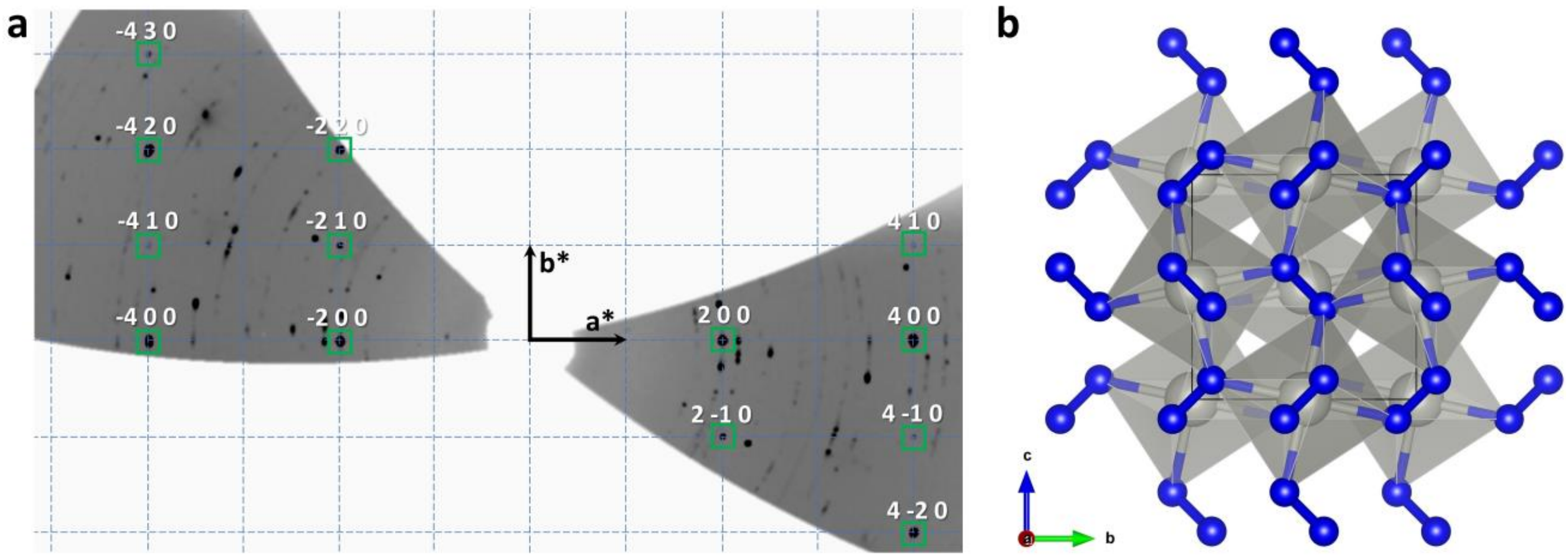

Figure SD1. (a) Reconstructed (*h k* 0) reciprocal lattice plane of PdN_2 at 102(2) GPa from the experimental single-crystal XRD dataset obtained using CrysAlis^{Pro} software. The green-labeled reflections correspond to the crystallite of PdN_2 whose structure was determined. Reflections (*h k* 0) with $h = 2n + 1$ are systematically absent in the *Pa*-3 space group. (b) Crystal structure of PdN_2 at 102(2) GPa, adopting the pyrite-type structure. The N-N bond length is 1.349(4) Å and Pd-N distance is 1.914(2) Å at 102(2) GPa. Pd atoms are grey, N atoms are blue.

Table SD2. The lattice parameters of selected PdN_2 grains at 102 and 118 GPa obtained from single crystal data sets.

P, GPa	102		118	
Unit cell	unconstrained	constrained	unconstrained	Constrained
a, Å	4.441(7)	4.444(2)	4.377(7)	4.399(3)
b, Å	4.443(5)	4.444(2)	4.410(9)	4.399(3)
c, Å	4.448(4)	4.444(2)	4.411(10)	4.399(3)
α, °	90.06(8)	90	90.49(18)	90
β, °	90.01(10)	90	90.45(16)	90
γ, °	90.22(12)	90	90.67(15)	90

Table SD3. The lattice parameters of PdN_2 at different pressures.

Beamline	Pressure, GPa	a, Å	Volume, Å^3
13ID-D	102(2)[1]	4.444(2)	87.77(12)
13ID-D	105(2)[1]	4.443(2)	87.728(6)
13ID-D	118(2)[1]	4.4141(6)	86.00(3)
HPCAT	113(2)	4.4240(4)	86.585(4)
HPCAT	109(2)	4.4343(4)	87.193(4)
HPCAT	101(2)	4.4492(4)	88.071(4)
HPCAT	93(2)	4.4603(5)	88.734(6)
HPCAT	78(2)	4.4921(6)	90.647(9)
HPCAT	64(2)	4.5387(5)	93.497(7)
HPCAT	41(2)[2]	4.6424(4)	100.055(6)

[1]Pressures at which laser-heating of the sample was performed.

[2]DAC was decompressed down to 41(2) GPa. At the next pressure point, one of the diamonds broke, and the pressure dropped to 1 atm. PdN_2 could not be found afterwards.

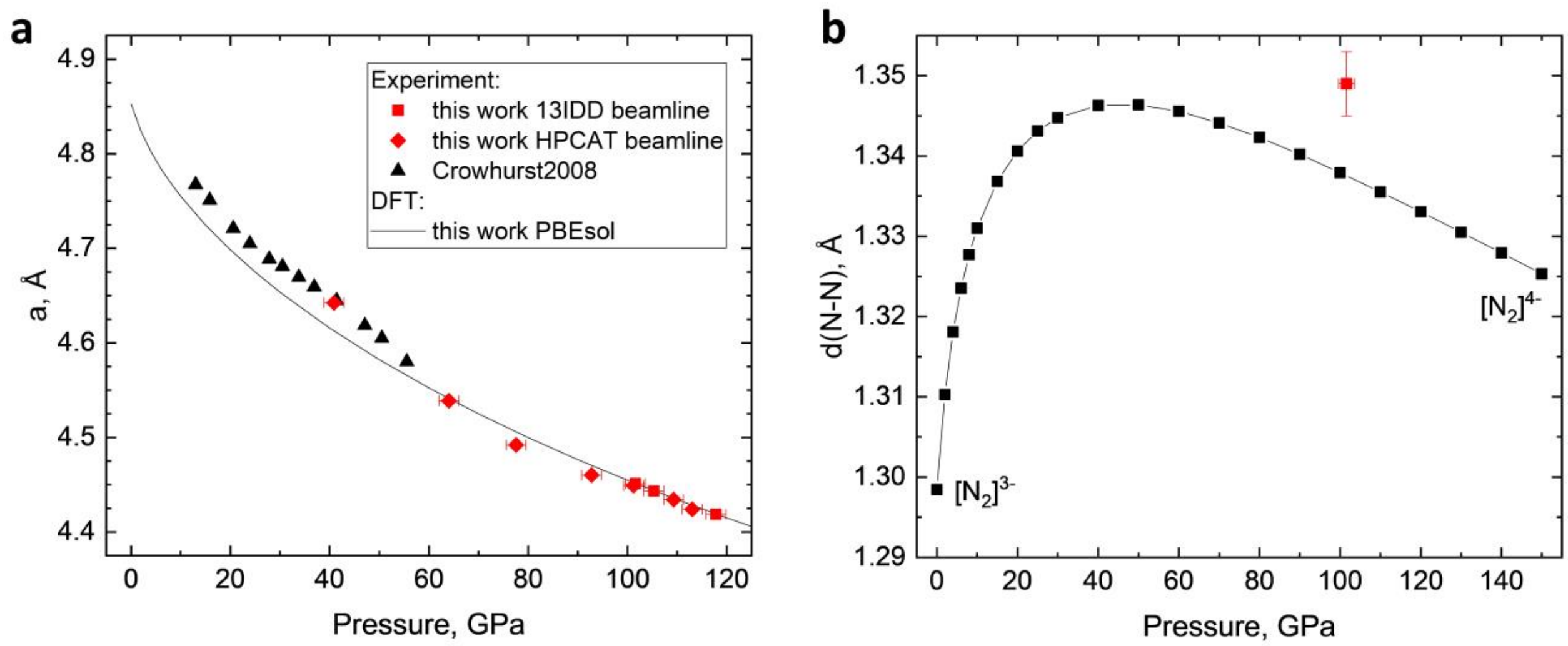

Figure SD2. (a) The pressure-lattice parameter dependence of PdN_2. (b) Calculated dependence of N-N bond length in PdN_2.

On decompression, the crystal structure remains triclinically distorted and the diffraction of PdN_2 was observed down to the lowest pressure we reached – 41(2) GPa (**Table SD3**). Pressure dependence of the cubic lattice parameter a of PdN_2 on decompression is consistent with the reported low-pressure data[1] (**Fig. SD2a**). We also run density functional theory (DFT) calculations using the PBEsol functional. While the calculated pressure-lattice parameter behavior shows no anomalies (**Fig. SD2a**), the N–N bond length exhibits unusual behavior during pressure release (**Fig. SD2b**). As the pressure decreases, the N–N bond length initially increases as expected, but below 40 GPa it decreases rapidly (**Fig. SD2b**). Analysis of the N–N bond length suggests that at elevated pressures the N–N bond corresponds to a single bond, consistent with the $[N_2]^{4-}$ pernitride anion and Pd^{4+}, whereas at ambient pressure the bond order is higher, corresponding to $[N_2]^{3-}$ and reduction Pd^{4+} to Pd^{3+}. Thus, our calculations indicate that pyrite-type PdN_2 undergoes a continuous phase transition starting around 40 GPa driven by an intrastructural redox reaction. Previous theoretical studies on PdN_2 did not report this behavior,[3] but similar behavior was experimentally observed in NiN_2.[4] Notably, PdN_2 is not recoverable experimentally,[1] implying that might be this phase transition lead to decomposition under real conditions.

Supplementary Tables

Table S1. Structure refinement details of $Pd(N_5)_2{\cdot}N_2$ at 118(2) GPa. The full crystallographic data were deposited to the ICSD under the deposition number CSD 2546222.

Chemical formula		$Pd(N_5)_2{\cdot}N_2$			
Temperature (K)		293			
Pressure (GPa)		118(2)			
**Crystal data**					
Mr		274.52			
ρ (g/cm^3)		5.732			
Crystal system, space group		orthorombic, *Pnnm*			
a (Å)		3.840(2)			
b (Å)		4.557(12)			
c (Å)		9.089(7)			
V (Å^3)		159.1(4)			
Z		2			
Radiation type		X-ray, λ = 0.29521 Å			
μ (mm^{-1})		3.219			
**Data collection**					
No. of measured, independent and observed [I > 2σ(I)] reflections		371/192/141			
R_{int}		4.35%			
$(\sin\theta/\lambda)_{max}$ (Å^{-1})		0.892			
**Refinement**					
R[F^2 > 4σ(F^2)], wR(F^2), GOF		5.86%, 13.68%, 1.19			
data/parameters ratio		192/18			
$\Delta\rho_{max}$, $\Delta\rho_{min}$ (e Å^{-3})		1.875, -1.993			
**Atomic positions and equivalent isotropic (or isotropic) ADPs**					
Atom	Wyckoff site	Fractional atomic coordinates			U_{iso} or U_{eq} (Å^2)
		x	y	z	
Pd1	2*b*	0	0	0.5	U_{eq} = 0.0066(5)
N1	4*f*	0	0.5	0.3569(10)	U_{iso} = 0.0051(17)
N2	8*h*	0.243(2)	0.406(3)	0.2734(9)	U_{iso} = 0.0058(14)
N3	8*h*	0.1560(19)	0.447(3)	0.1427(8)	U_{iso} = 0.0036(15)
N4	4*g*	0.058(3)	0.891(5)	0	U_{iso} = 0.008(2)

Table S2. The lattice parameters of $Pd(N_5)_2{\cdot}N_2$ at different pressures.

Beamline	Pressure, GPa	a, Å	b, Å	c, Å	Volume, Å^3
13ID-D	118(2)[1]	3.840(2)	4.557(12)	9.089(7)	159.1(4)
HPCAT	113(2)	3.836(2)	4.556(2)	9.140(9)	159.7(2)
HPCAT	109(2)	3.852(2)	4.583(3)	9.146(7)	161.4(2)
HPCAT	101(2)	3.870(1)	4.596(2)	9.189(5)	163.4(1)
HPCAT	93(2)	3.884(1)	4.631(2)	9.246(7)	166.3(2)
HPCAT	78(2)	3.935(4)	4.681(6)	9.330(16)	171.9(4)
HPCAT	64(2)	3.988(3)	4.775(8)	9.508(15)	181.1(4)

[1]Pressure of synthesis.

Table S3. Experimentally determined crystallographic data for $Pd(N_5)_2 \cdot N_2$ phase at 118 GPa in comparison with the corresponding DFT-relaxed structure. Note that pressure was fixed in theoretical simulations, while volumes of the unit cells, lattice parameters and equilibrium state parameters were calculated.

		Experiment			Theory		
**Pressure**		118(2) GPa			118 GPa (fixed)		
**Space group**		*Pnnm*			*Pnnm*		
**Volume**		159.1(4) Å^3			156.7 Å^3		
**Lattice parameters**		a = 3.840(2) Å<br>b = 4.557(12) Å<br>c = 9.089(7) Å			a = 3.8204 Å<br>b = 4.5081 Å<br>c = 9.0990 Å		
**Atom positions**							
**Atom**		**x**	**y**	**z**	**x**	**y**	**z**
**Pd1**		0	0	0.5	0	0	0.5
**N1**		0	0.5	0.3569(10)	0	0.5	0.3579
**N2**		0.243(2)	0.406(3)	0.2734(9)	0.2467	0.4068	0.2744
**N3**		0.1560(19)	0.447(3)	0.1427(8)	0.1571	0.4462	0.1431
**N4**		0.058(3)	0.891(5)	0	0.0615	0.8894	0
**Bond length**							
**N_5 ring**	**N1-N2**	1.292(17) Å			1.282 Å		
	**N2-N3**	1.248(12) Å			1.256 Å		
	**N3-N3**	1.292(17) Å			1.295 Å		
**N_2 dimer**	**N4-N4**	1.09(5) Å			1.102 Å		

Table S4. Integrated crystal orbital bond index for Pd-N and N-N contacts/bonds in $Pd(N_5)_2 \cdot N_2$ at 118 GPa.

Contacts/bonds		Distance, Å	ICOBI
Pd-N contacts/bonds	Pd-N1	2.598	0.03
	Pd-N2	2.711	0.02
	Pd-N3	1.862	0.39
	Pd-N4	2.427	0.04
N_5 ring	N1-N2	1.282	1.32
	N2-N3	1.256	1.35
	N3-N3	1.295	1.30
N_2 dimer	N4-N4	1.102	2.54

Table S5. Charge distribution analysis in $Pd(N_5)_2 \cdot N_2$ at 118 GPa.

Atom/unit	Bader charge	Mulliken charge	Löwdin charge
Pd atom	0.99	1.10	0.84
N_5 ring	-0.42	-0.45	-0.34
N_2 dimer	-0.15	-0.2	-0.16

Table S6. Summary of cation properties, reported pentazolate compounds, synthesis P–T conditions, and structural characterization methods.

**Metal**	**Oxidation state**	**Electro negativity**	**Ionic radius, Å**	**Observed compounds, reference**	**Synthesis pressure, GPa**	**Synthesis temperature, K**	**Characterization method**
**Y**	+3	1.22	1.08 ^{IX}	$Y(N_5)_3{\cdot}N_2$ [5]	125	3000	SCXRD
**Pd**	+2	2.20	0.64 ^{IVsq}	$Pd(N_5)_2{\cdot}N_2$	118	1900	SCXRD
**Ag**	+1	1.93	1.00 ^{IV}	AgN_5 [6]	15	Not reported	CSP+PXRD
				$AgN_5{\cdot}N_2$ [6]	15	Not reported	CSP+PXRD
				AgN_5 [7]	118	2000	SCXRD
**Li**	+1	0.98	0.76 ^{VI}	LiN_5 [8]	41.1	Not reported	CSP+PXRD
				LiN_5 [9]	45	Not reported	PXRD
				LiN_5 [10]	60	2000	CSP+PXRD
**Na**	+1	0.93	1.24 ^{IX}	NaN_5 [11]	19.6	Not reported	CSP+PXRD
				NaN_5 [12]	52	2200	SCXRD
				$NaN_5{\cdot}N_2$ [12]	52	2200	SCXRD
**K**	+1	0.82	1.64 ^{XII}	K_9N_{56} [13]	46	>2000	SCXRD
**Cs**	+1	0.79	1.88 ^{XII}	CsN_5 [14]	60	Not reported	CSP+PXRD

Notes: Electronegativity is given in Pauling scale; Ionic radius is provided for coordination number given in superscript; SCXRD – single crystal X-ray diffraction from multigrain sample; PXRD – powder X-ray diffraction; CSP – crystal structure prediction;

Supplementary Figures

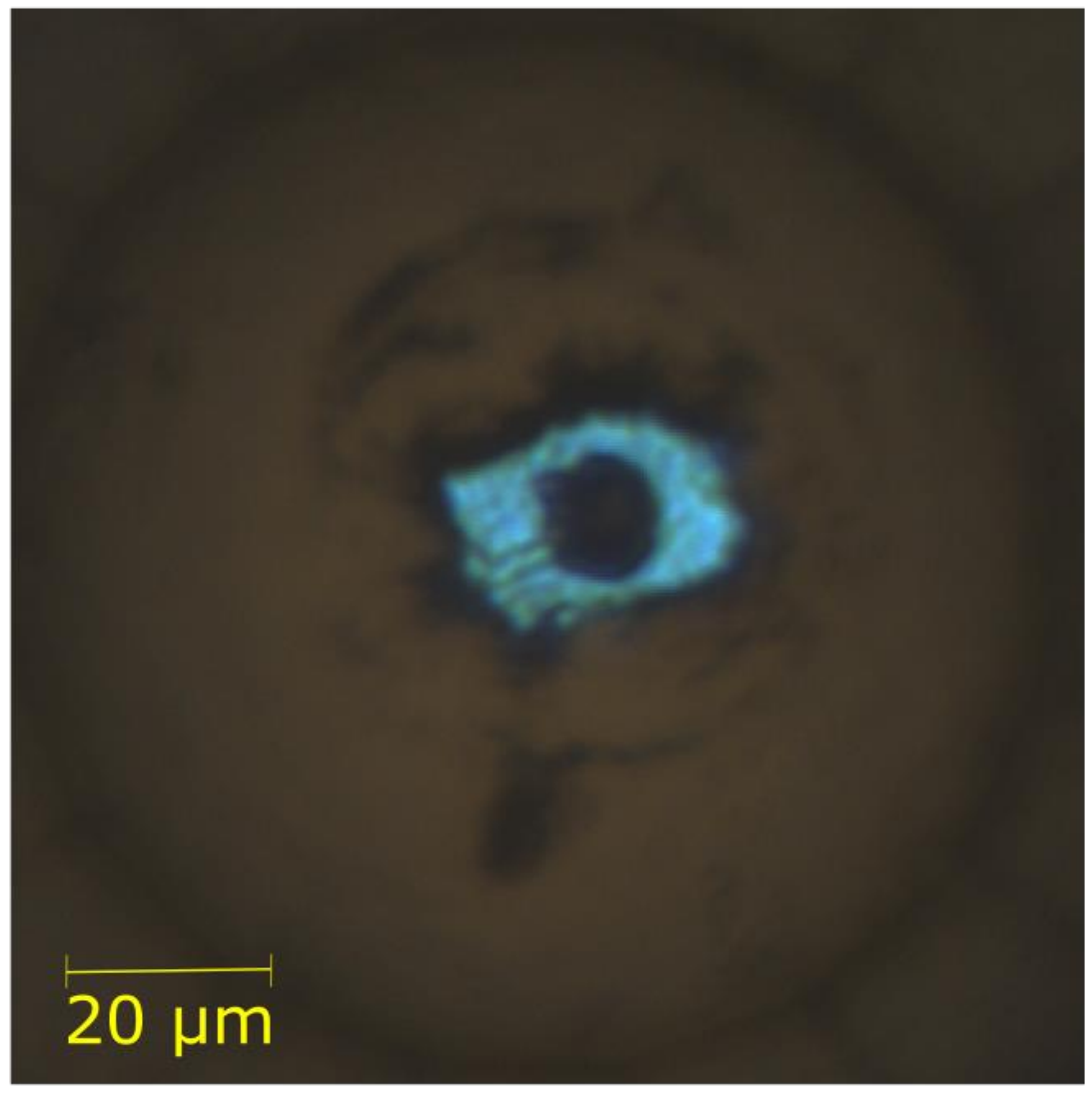

Figure S1. Microphotograph of the sample chamber loaded with a piece of Pd foil and nitrogen.

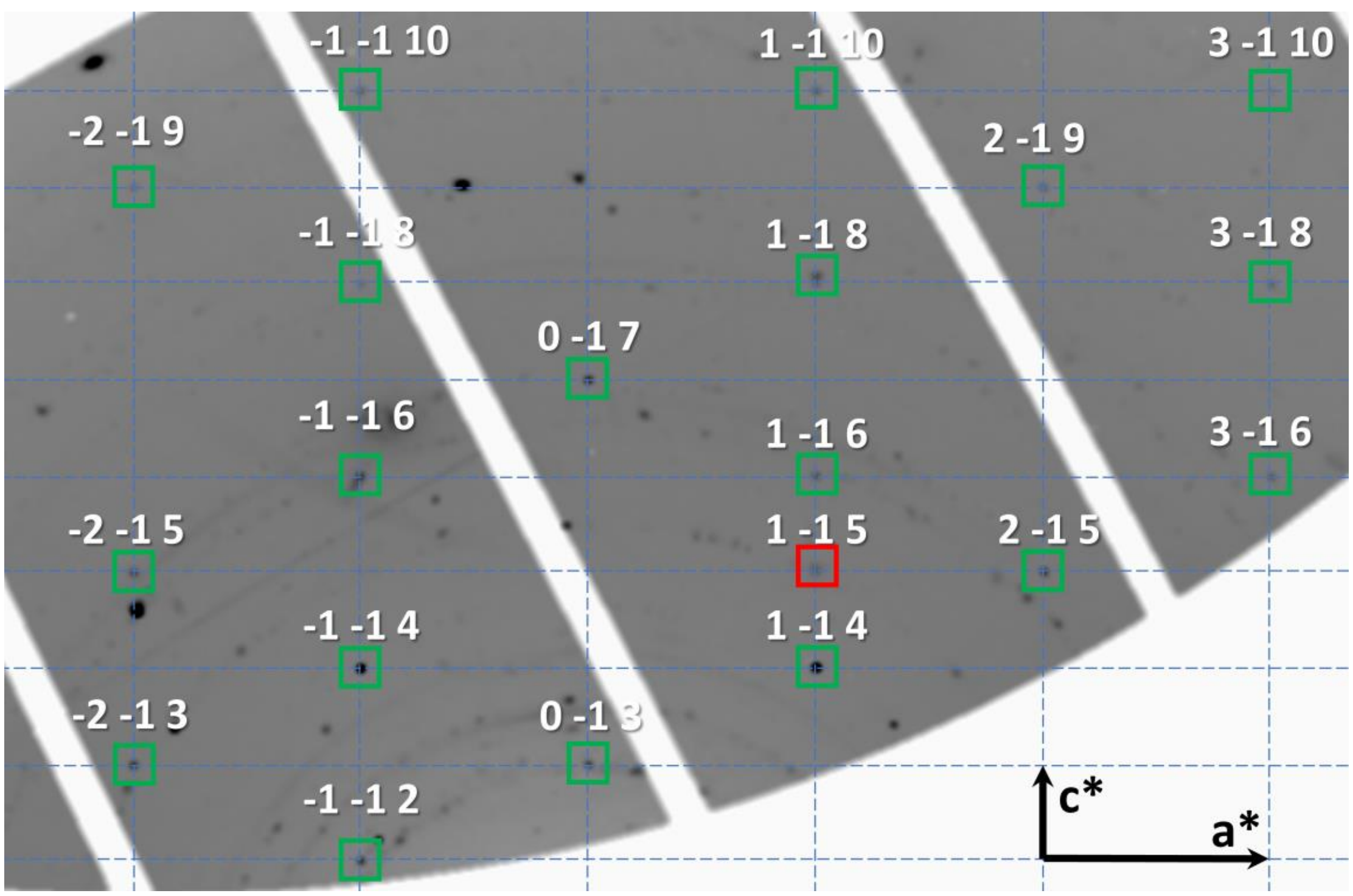

Figure S2. Part of the reconstructed (*h* -1 *l*) reciprocal lattice plane of $Pd(N_5)_2 \cdot N_2$ at 118(2) GPa from the experimental single-crystal XRD dataset obtained using CrysAlis^{Pro} software. Green labels mark reflections with $h+k+l = 2n$, whose intensities include contributions from the Pd atoms. A red label marks a reflection with $h+k+l = 2n+1$, whose intensity arise solely from the N2 and N3 atoms. The presence of reflections with $h+k+l = 2n+1$ confirms that the unit cell is primitive rather than *I*-centered.

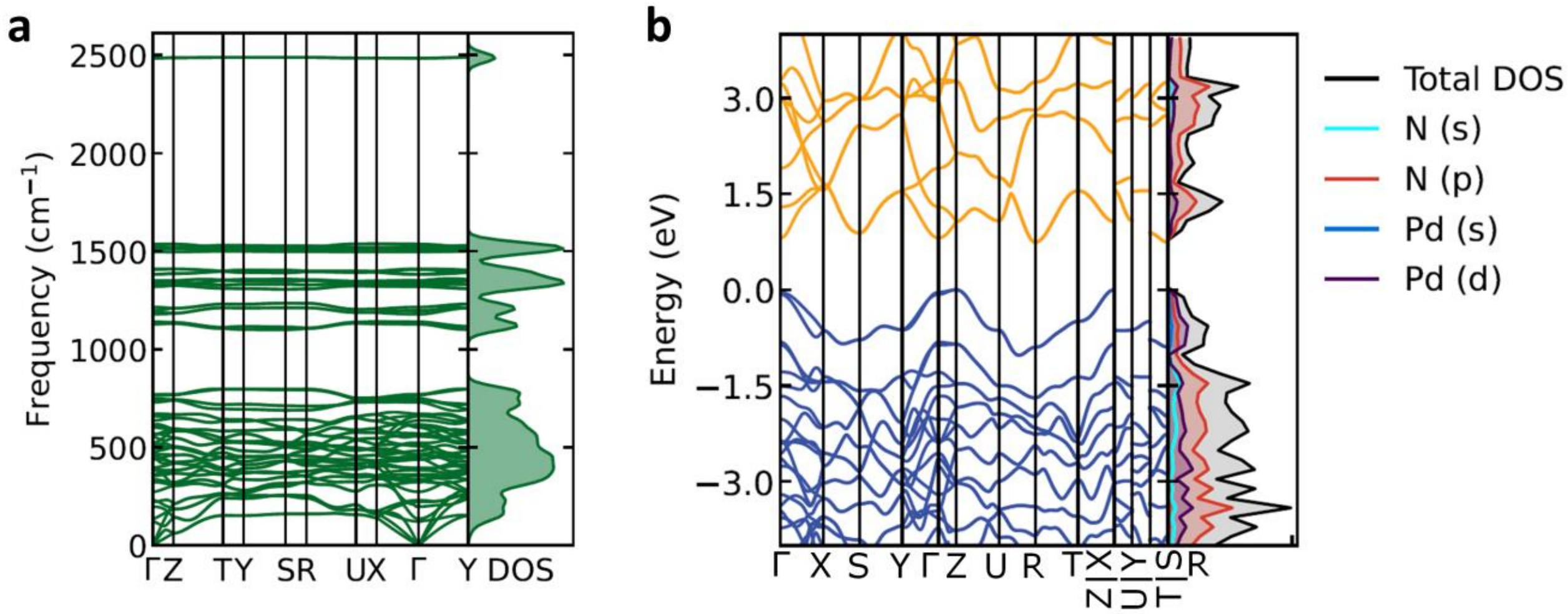

Figure S3. (a) Phonon dispersion and (b) band structure of $Pd(N_5)_2{\cdot}N_2$ at 118 GPa.

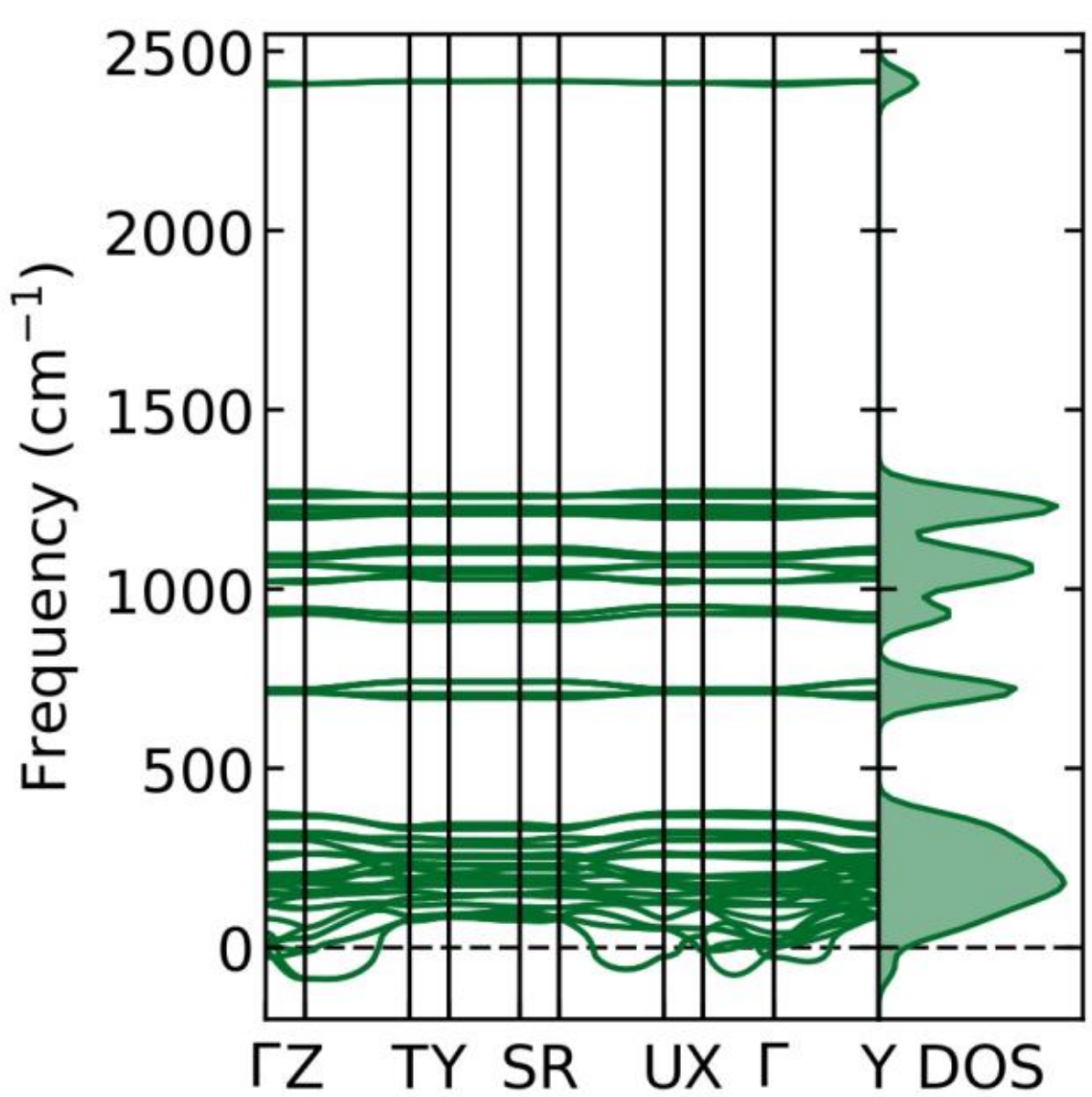

Figure S4. Phonon dispersion of $Pd(N_5)_2{\cdot}N_2$ at 1 bar. The presence of imaginary modes demonstrates its dynamical instability at ambient pressure.

References

(1) Crowhurst, J. C.; Goncharov, A. F.; Sadigh, B.; Zaug, J. M.; Aberg, D.; Meng, Y.; Prakapenka, V. B. Synthesis and Characterization of Nitrides of Iridium and Palladium. *J. Mater. Res.* **2008**, *23* (1), 1–5. https://doi.org/10.1557/jmr.2008.0027.

(2) Bayliss, P. Crystal Structure Refinement of a Weakly Anisotropic Pyrite. *Am. Mineral.* **1977**, *62* (11–12), 1168–1172.

(3) Åberg, D.; Erhart, P.; Crowhurst, J.; Zaug, J. M.; Goncharov, A. F.; Sadigh, B. Pressure-Induced Phase Transition in the Electronic Structure of Palladium Nitride. *Phys. Rev. B* **2010**, *82* (10), 104116. https://doi.org/10.1103/PhysRevB.82.104116.

(4) Bykov, M.; Bykova, E.; Ponomareva, A. V.; Tasnádi, F.; Chariton, S.; Prakapenka, V. B.; Glazyrin, K.; Smith, J. S.; Mahmood, M. F.; Abrikosov, I. A.; Goncharov, A. F. Realization of an Ideal Cairo Tessellation in Nickel Diazenide NiN_2: High-Pressure Route to Pentagonal 2D Materials. *ACS Nano* **2021**, *15* (8), 13539–13546. https://doi.org/10.1021/acsnano.1c04325.

(5) Aslandukov, A.; Yin, Y.; Bykov, M.; Aslandukova, A.; Akbar, F. I.; Bright, E. L.; Abrikosov, I. A.; Dubrovinskaia, N.; Dubrovinsky, L. High-Pressure Synthesis of Nitrogen-Rich $Y(N_5)_3{\cdot}N_2$ Pentazolate with Perovskite Topology. *Angew. Chemie Int. Ed.* **2025**, e202506334. https://doi.org/10.1002/anie.202506334.

(6) Zhang, H.; Liu, R.; Yuan, J.; Wang, D.; Liu, R.; Wang, Y.; Liu, Z.; Yao, Z.; Zhang, Y.; Liu, S.; Wang, P. Stabilized N_5 and N_6 Rings in the Ag–N System under Modest Pressure. *Matter Radiat. Extrem.* **2025**, *10* (May), 067801. https://doi.org/10.1063/5.0282196.

(7) Liang, A.; Eikelder, H. R. A.; Ranieri, U.; Spender, J.; Massani, B.; Fedotenko, T.; Glazyrin, K.; Giordano, N.; Bright, E. L.; Wright, J.; Shi, L.; Trybel, F.; Laniel, D. High-Pressure Synthesis of the First Thermodynamically Stable Silver Nitride, AgN_5. *JACS Au* **2026**, *6* (1), 193–199. https://doi.org/10.1021/jacsau.5c01135.

(8) Zhou, M.; Sui, M.; Shi, X.; Zhao, Z.; Guo, L.; Liu, B.; Liu, R.; Wang, P.; Liu, B. Lithium Pentazolate Synthesized by Laser Heating-Compressed Lithium Azide and Nitrogen. *J. Phys. Chem. C* **2020**, *124* (22), 11825–11830. https://doi.org/10.1021/acs.jpcc.0c03949.

(9) Laniel, D.; Weck, G.; Gaiffe, G.; Garbarino, G.; Loubeyre, P. High-Pressure Synthesized

Lithium Pentazolate Compound Metastable under Ambient Conditions. *J. Phys. Chem. Lett.* **2018**, *9* (7), 1600–1604. https://doi.org/10.1021/acs.jpclett.8b00540.

(10) Zhao, Z.; Liu, R.; Guo, L.; Liu, S.; Sui, M.; Niu, S.; Liu, B.; Wang, P.; Yao, Z.; Liu, B. High-Pressure Synthesis and Stability Enhancement of Lithium Pentazolate. *Inorg. Chem.* **2022**, *61* (24), 9012–9018. https://doi.org/10.1021/acs.inorgchem.2c00112.

(11) Zhou, M.; Liu, S.; Du, M.; Shi, X.; Zhao, Z.; Guo, L.; Liu, B.; Liu, R.; Wang, P.; Liu, B. High-Pressure-Induced Structural and Chemical Transformations in NaN_3. *J. Phys. Chem. C* **2020**, *124* (37), 19904–19910. https://doi.org/10.1021/acs.jpcc.0c04107.

(12) Bykov, M.; Bykova, E.; Chariton, S.; Prakapenka, V. B.; Batyrev, I. G.; Mahmood, M. F.; Goncharov, A. F. Stabilization of Pentazolate Anions in the High-Pressure Compounds Na_2N_5 and NaN_5 and in the Sodium Pentazolate Framework $NaN_5 \cdot N_2$. *Dalt. Trans.* **2021**, *50* (21), 7229–7237. https://doi.org/10.1039/d1dt00722j.

(13) Laniel, D.; Trybel, F.; Yin, Y.; Fedotenko, T.; Khandarkhaeva, S.; Aslandukov, A.; Aprilis, G.; Abrikosov, A. I.; Bin Masood, T.; Giacobbe, C.; Bright, E. L.; Glazyrin, K.; Hanfland, M.; Wright, J.; Hotz, I.; Abrikosov, I. A.; Dubrovinsky, L.; Dubrovinskaia, N. Aromatic Hexazine $[N_6]^{4-}$ Anion Featured in the Complex Structure of the High-Pressure Potassium Nitrogen Compound K_9N_{56}. *Nat. Chem.* **2023**, *15* (5), 641–646. https://doi.org/10.1038/s41557-023-01148-7.

(14) Steele, B. A.; Stavrou, E.; Crowhurst, J. C.; Zaug, J. M.; Prakapenka, V. B.; Oleynik, I. I. High-Pressure Synthesis of a Pentazolate Salt. *Chem. Mater.* **2017**, *29* (2), 735–741. https://doi.org/10.1021/acs.chemmater.6b04538.
